# New Delhi metallo-β-lactamase 1 [nū del′ē mә-tal′ō bāt′ә lak′tә-mās wuhn]

**DOI:** 10.3201/eid3206.241434

**Published:** 2026-06

**Authors:** Surajit Chakraborty

**Affiliations:** All India Institute of Medical Sciences, Bathinda, India

**Keywords:** New Delhi metallo-β-lactamase 1, NDM-1, carbapenem resistance, transmissible genetic element, Ambler class B, bacteria, antimicrobial resistance

New Delhi metallo-β-lactamase 1 (NDM-1) is an Ambler class B β-lactamase enzyme named after the capital of India, New Delhi. This class of enzymes requires Zn2+ ions for activity, hence metallo-β-lactamase. The transmissible genetic element–associated *bla*_NDM-1_ encodes NDM-1, which confers resistance to all β-lactam antibiotics except monobactams. The gene was first identified and reported in 2009 from a urinary tract infection–causing carbapenem-resistant *Klebsiella pneumoniae* isolate. Reportedly, the *bla*_NDM-1_–harboring *K. pneumoniae* strain (linked to sequence type 14) was isolated from an India-born patient in Sweden who acquired a urinary tract infection while visiting New Delhi. 

NDM’s eponymous association with New Delhi sparked anguish among authorities in India, who saw the terminology as a means to tarnish the country’s growing medical tourism industry. Some suggested changing the term to PCM (plasmid-encoding carbapenem-resistant metallo-β-lactamase). However, the sporadic concerns regarding nomenclature were never formally addressed, and the term NDM-1 eventually gained universal acceptance within the scientific community. By the end of 2025, >63 distinct NDM variants had been reported worldwide and sequentially designated NDM-1 through NDM-63.
